# Barriers, Facilitators, and Requirements for a Telerehabilitation Aftercare Program for Patients After Occupational Injuries: Semistructured Interviews With Key Stakeholders

**DOI:** 10.2196/51865

**Published:** 2024-11-08

**Authors:** Lukas Lange-Drenth, Holger Schulz, Isabell Suck, Christiane Bleich

**Affiliations:** 1 Department of Medical Psychology University Medical Center Hamburg-Eppendorf Hamburg Germany

**Keywords:** telerehabilitation, rehabilitation, eHealth development, value specification, stakeholder participation, occupational injuries, vocational rehabilitation, aftercare, mobile phone

## Abstract

**Background:**

Patients with occupational injuries often receive multidisciplinary rehabilitation for a rapid return to work. Rehabilitation aftercare programs give patients the opportunity to help patients apply the progress they have made during the rehabilitation to their everyday activities. Telerehabilitation aftercare programs can help reduce barriers, such as lack of time due to other commitments, because they can be used regardless of time or location. Careful identification of barriers, facilitators, and design requirements with key stakeholders is a critical step in developing a telerehabilitation aftercare program.

**Objective:**

This study aims to identify barriers, facilitators, and design requirements for a future telerehabilitation aftercare program for patients with occupational injuries from the perspective of the key stakeholders.

**Methods:**

We used a literature review and expert recommendations to identify key stakeholders. We conducted semistructured interviews in person and via real-time video calls with 27 key stakeholders to collect data. Interviews were transcribed verbatim, and thematic analysis was applied. We selected key stakeholder statements about facilitators and barriers and categorized them as individual, technical, environmental, and organizational facilitators and barriers. We identified expressions that captured aspects that the telerehabilitation aftercare program should fulfill and clustered them into attributes and overarching values. We translated the attributes into one or more requirements and grouped them into content, functional, service, user experience, and work context requirements.

**Results:**

The key stakeholders identified can be grouped into the following categories: patients, health care professionals, administrative personnel, and members of the telerehabilitation program design and development team. The most frequently reported facilitators of a future telerehabilitation aftercare program were time savings for patients, high motivation of the patients to participate in telerehabilitation aftercare program, high usability of the program, and regular in-person therapy meetings during the telerehabilitation aftercare program. The most frequently reported barriers were low digital affinity and skills of the patients and personnel, patients’ lack of trust and acceptance of the telerehabilitation aftercare program, slow internet speed, program functionality problems (eg, application crashes or freezes), and inability of telerehabilitation to deliver certain elements of in-person rehabilitation aftercare such as monitoring exercise performance. In our study, the most common design requirements were reducing barriers and implementing facilitators. The 2 most frequently discussed overarching values were tailoring of telerehabilitation, such as a tailored exercise plan and tailored injury-related information, and social interaction, such as real-time psychotherapy and digital and in-person rehabilitation aftercare in a blended care approach.

**Conclusions:**

Key stakeholders reported on facilitators, barriers, and design requirements that should be considered throughout the development process. Tailoring telerehabilitation content was the key value for stakeholders to ensure the program could meet the needs of patients with different types of occupational injuries.

## Introduction

### Background

The International Labour Organization estimates that each year there are 313 million nonfatal occupational accidents worldwide that result in at least 4 day of absence from work [1]. In 2021, there were 2.9 million nonfatal occupational accidents in the European Union that resulted in at least 4 calendar days of absence from work [2]. The upper extremities were the body parts most frequently affected by occupational injuries or illnesses in the United States (284,860/888,220, 32.07%) and Germany (374,557/780,581, 47.98%) in 2019 [3,4].

Traumatic occupational injuries can have a psychological impact on workers [5,6]. Posttraumatic stress disorder appears to be the most commonly reported mental disorder following occupational injury [5] and is negatively associated with return to work [7]. The prevalence of posttraumatic stress disorder in patients hospitalized for ≥3 days after an occupational injury is estimated to be 5% at 12 months [8] and 7% at 6 years [9].

In 2020, 7.59% (65,599/863,734) of the German patients who had an occupational accident received outpatient or inpatient rehabilitation after acute treatment [10]. In Germany, all medical treatment of patients after occupational accidents is covered and organized by the German statutory accident insurance [11,12]. The rapid reintegration of the patients into the work process after an occupational accident is the central goal of the rehabilitation [11,13]. Returning to work is important because it is associated with improved self-reported health, self-esteem, health-related quality of life, reduced distress, fewer depressive symptoms, and fewer financial worries [14,15]. German patients who are severely injured with long-term impairments are assigned a personal contact—the rehabilitation manager. The rehabilitation manager plans and coordinates the rehabilitation and services necessary for participation in work or social life in cooperation with the patients and all parties involved in the process [16]. The basis for the rehabilitation management of the German statutory accident insurance is the International Classification of Functioning, Disability and Health [17], which, with its biopsychosocial perspective, provides a systematic approach that supports a holistic view of workers who are injured [11]. After acute treatment, German workers who are injured receive multidisciplinary inpatient or outpatient rehabilitation programs or outpatient monotherapy programs, which can vary widely depending on the type of injury, functional impairment, job requirements, and environmental factors [11,13]. The multidisciplinary rehabilitation programs combine treatment elements such as occupational therapy, functional and work capacity assessments, physiotherapy, mechanotherapy, speech therapy for central or peripheral nervous system injuries, and psychotherapy [11]. Systematic reviews show that these types of multidisciplinary vocational rehabilitation services can improve employment outcomes and short-term functional outcomes for conditions commonly resulting from occupational injuries, such as spinal cord and brain injuries; back pain; and musculoskeletal conditions of the forearm, wrist, and hand [18-20]. However, the methodological quality of the studies included in the systematic reviews was low to moderate [18-20]. In addition, no randomized controlled trials have yet evaluated the effectiveness of vocational rehabilitation in helping patients with upper extremity injuries—the body part most commonly affected by occupational injuries—return to work [21].

The success and sustainability of rehabilitation programs depend to a large extent on how well rehabilitants are able to transfer and integrate what they have learned in rehabilitation into their everyday lives and maintain those changes over time [22]. After returning to work following orthopedic rehabilitation, patients with severe limitations in work-related functioning reported strong interest in additional work-related functional capacity training, work-related psychosocial groups, and social counseling [23]. Furthermore, aftercare programs that continue the initial intensive rehabilitation treatment show promising results in maintaining rehabilitation success. Results from a multicenter study indicate that work-related aftercare and conventional aftercare following outpatient treatment in rehabilitation centers have contributed to substantial improvements in the quality of life and work ability in patients with severe work limitations from baseline to 6 months [24] and 12 months after the start of aftercare [25].

Telerehabilitation programs can help reduce external and motivational barriers to participation in exercise-based rehabilitation and aftercare programs for patients after occupational accidents. In our opinion, one of the most important barriers that can be reduced by telerehabilitation is the lack of time due to commitments to other priorities, such as work and family [26,27]. Telerehabilitation programs can be used regardless of time and place. Exercise-based telerehabilitation programs, such as exercise instructions through a digital avatar [28-30] or video [31-36], games on the Nintendo Wii (Nintendo Co Ltd) [37,38] or a smartphone [39], outpatient physical therapy combined with serious games [40], exergames using automated sensor-based technology [41], home exercise programs with additional mobile health–based education [42] or with additional motivational SMS text messaging [43], smartphone-assisted home exercise program [35], home exercise with asynchronous telemonitoring [44], and real-time therapy via web-based video calls [45], have shown comparable outcomes for pain [30,31,35,36,38,46,47], physical functioning [29,34-37,40-44,46-48], mental health [29-31], and physical health [29-31] compared with outpatient physical therapy [28-31,34,38-41,44,46], home exercise [32,35,37,42,43,48] programs, and verbal exercise education and discharge education [36] for the rehabilitation of patients with orthopedic injuries and disorders. In addition, telerehabilitation programs had higher adherence rates than outpatient physical therapy programs [30,33] or home exercise programs [43,48] for patients after total knee arthroplasty (TKA) [30], patients with upper or lower limb musculoskeletal conditions [48], and patients recovering from flexor tendon repair [33,43]. In addition, 2 randomized controlled trials showed that a digital self-management application [49] and an internet-based cognitive behavioral therapy [50] showed greater reductions in posttraumatic stress and depressive symptoms [49] and posttraumatic symptoms [50] compared with waitlist controls for patients with posttraumatic stress disorder.

Although the results of these telerehabilitation programs are promising, there are various barriers and facilitators to the implementation and adoption of eHealth programs in general [51], real-time web-based consultations for patients with various health conditions [52], and synchronous telerehabilitation specifically for patients with musculoskeletal disorders [53]. These barriers and facilitators can be categorized into three groups: (1) individual, (2) environmental and organizational, and (3) technical [54]. The most frequently cited individual, environmental and organizational, and technical barriers and facilitators were (1) limited digital affinity and skills, low user motivation [51], resistance to technology [52], privacy concerns, and lack of personal contact [52,53] on the one hand and high user motivation [51,52], time savings, and satisfaction with the program [52,53] on the other hand; (2) technical issues such as poor audio or video quality, program functionality problems [52,53], and internet speed [52] on the one hand and ease of use, high audio and video quality [51-53], and patient training [52] on the other hand; and (3) financial issues on the one hand and cost-effectiveness compared to analogue care on the other hand [52,53].

To develop meaningful and effective eHealth technologies, the needs, barriers, and facilitators of key stakeholders should be identified and translated into specific requirements before the design phase of a new eHealth technology begins [55-60]. Key stakeholders are those who are most affected by the potential eHealth program and whose views are, therefore, of great importance for design and implementation [57,61]. A recent review highlighted the importance of formulating concrete requirements for monitoring systems in dementia care so that they can be used by technology developers and are based on the perspective of those who might use them [62]. This study was based on the comprehensive roadmap developed by the Center for e-Health Research and Disease Management (CeHRes) [57]. The CeHRes roadmap is a holistic broad-based approach that engages key stakeholders in the design and development of sustainable eHealth interventions [56,57,60] and consists of the following iterative phases: contextual inquiry, value specification, design, operationalization, and summative evaluation [57]. This study focused on the contextual inquiry and value specification phases. During the contextual inquiry phase, key stakeholders and their views on the current situation, its weaknesses and strengths, and stakeholders’ needs should be identified to determine whether and in what way an eHealth technology can contribute to the current situation. In the value specification phase, these insights can be used to formulate the value a technology could add to the current situation. The values can then be translated into specific requirements for the eHealth technology [56,57]. These requirements describe the design details, specifically, what a technology should do, what content it should display, and what type of user experience it should provide [56].

### Objectives

The overall objective of this study was to provide insight into the contextual inquiry and value specification phases of the development process of a future telerehabilitation aftercare program for patients after occupational accidents following initial in-person rehabilitation. The following research questions were addressed: (1) What are the barriers and facilitators to a future telerehabilitation aftercare program according to key stakeholders? and (2) What are the design requirements for a future telerehabilitation aftercare program?

## Methods

### Study Design

We used an exploratory qualitative research design. We conducted semistructured interviews in person and via real-time video call with 27 key stakeholders to gain insight into their views on the current rehabilitation of patients with occupational injuries. We also explored the potential benefits that a telerehabilitation aftercare technology could offer in a context where patients currently receive in-person rehabilitation.

This study follows the recommendations of the Standards for Reporting Qualitative Research, which consists of 21 items aiming to improve the transparency of all aspects of qualitative research by providing clear standards for reporting qualitative research (Multimedia Appendix 1) [63].

### Stakeholder Analysis

We used a literature review and expert recommendations to identify stakeholders and key stakeholders [61]. First, we listed all probable stakeholders by reviewing randomized controlled trials of telerehabilitation programs for patients with upper or lower extremity trauma injuries [28-33,37-39,46-48]. Second, a group of experts in the field discussed and adjusted the list. The group consisted of the following participants: (1) the first author of this study, who has several years of experience in eHealth research, (2) the medical director of an eHealth provider in rehabilitation, and (3) 2 quality assurance consultants of the largest statutory accident insurer in Germany. Disagreements among the experts regarding the importance of stakeholders’ views on the telerehabilitation aftercare program were resolved through discussion.

### Setting, Participants, and Recruitment

We recruited participants between January and May 2021. The recruitment process differed for the patients and the personnel. The clinic site managers recruited the patients at 1 outpatient and 1 inpatient rehabilitation clinic. The clinic site managers approached the patients during their visits or stays at the clinics. The medical director of an eHealth provider in rehabilitation and the 2 quality assurance consultants of the statutory accident insurance provider recruited the other participants. Patients were included if they were undergoing treatment for their occupational injury and had sufficient oral and written German language skills. We deliberately selected a sample that was as heterogeneous as possible in terms of age, digital skills, German language skills, and severity of injury to reflect heterogeneous views on a future telerehabilitation aftercare program. Personnel were included if they had >1 year of professional experience in the care of patients after occupational accidents or in administrative work for patients after occupational accidents and if they had sufficient oral and written German language skills. In addition, personnel were recruited only from those clinics that had already worked with telerehabilitation programs but had not used telerehabilitation for patients whose rehabilitation was covered by statutory accident insurance. We scheduled an appointment for the semistructured interviews within the following 3 weeks. The clinic site manager gave an informed consent form to all participants, or they received it by email. The informed consent form included information about the purpose of the study, the potential risks and benefits of the study, the voluntary nature of participation, and the nature and duration of data storage. Four patient interviews were conducted in a treatment room of an outpatient rehabilitation clinic; 3 patient interviews and all interviews with personnel were conducted on the web via Zoom (Zoom Video Communications, Inc). In the treatment rooms, the interviewer and the interviewee sat on 2 chairs with a table in between them, on which a mobile phone was placed as an audio recording device. In the Zoom meetings, it was ensured that the interviewer and the interviewee sat in a room by themselves to minimize distractions.

### Semistructured Interviews

The in-person and real-time video call interviews were guided by study-specific interview topic guides (Multimedia Appendix 2) [64], which were inspired by interview topic guides from previous studies [60,65]. During the data collection process, we iteratively supplemented the initial topic guide to effectively encompass areas of specific importance. There were two versions of the topic guide: one for the patients and another for the health care professionals, members of the design and development team, and administrative personnel. The main topics of the patient interviews were as follows: (1) sociodemographic and medical characteristics and internet use, (2) grand tour question and facilitators of a telerehabilitation aftercare program, (3) current situation of rehabilitation after occupational accidents, (4) facilitators and requirements of a telerehabilitation aftercare program, and (5) barriers to a telerehabilitation aftercare program. The main topics of the interviews with health care professionals, design and development team members, and administrative personnel were as follows: (1) sociodemographic and work-related characteristics, (2) grand tour question and requirements for a telerehabilitation aftercare program, (3) current situation of telerehabilitation programs, (4) individual facilitators and barriers and the added value of a telerehabilitation aftercare program, (5) environmental and organizational barriers and facilitators, and (6) technical barriers and facilitators and requirements for a telerehabilitation aftercare program. All interviews were conducted by a trained interviewer (LL-D) and recorded using Open Broadcaster software (open source, Hugh Bailey, version 27.0.1), which generated audio data. Interviews lasted an average of 27.5 (SD 6.2; range 17-40) minutes. Participants were included in the study until saturation was reached.

### Data Analysis

The audiotapes of the interviews were transcribed verbatim, and thematic analysis, inspired by the studies by Braun and Clarke [66,67], was applied using qualitative data analysis software MAXQDA (version 20.3.0, VERBI software). We used a hybrid approach in our thematic analysis, starting with predefined codes based on existing literature, such as barriers and facilitators. However, within these broad categories, themes and subthemes were allowed to emerge inductively from the data, trying to ensure that the analysis remained grounded in participants’ actual experiences.

First, we selected relevant key stakeholder statements about facilitators and barriers of a telerehabilitation aftercare program, which we first coded (initial codes) and categorized into individual, technical, environmental, and organizational facilitators and barriers as defined by Griebel et al [54]. We further inductively grouped selected fragments within the 6 categories into overarching themes and subthemes and named these subthemes. To control for selectivity during the coding process, a second analyst (IS) independently coded 5 (18.5%) of the 27 interviews of the data. This independent coding by IS served as a means of cross-checking the initial interpretations and identifying any potential biases introduced by the first coder. The 2 researchers used the coded transcript in MAXQDA and compared which statements they had coded and the codes they had assigned. They also discussed whether the identified themes and subthemes were truly representative of the data. Differences in codes, themes, and subthemes were resolved through discussion, with the final coding being agreed upon by consensus.

We identified requirements for a telerehabilitation aftercare program for patients after occupational accidents based on the procedures described by Van Velsen et al [68]. We began by identifying and grouping key stakeholder comments that highlighted the aspects the telerehabilitation aftercare program should address into specific attributes and overarching values. We defined an attribute as “a summary of the need or that is spoken out by the (future) end user or stakeholder” [68]. Second, we translated the attributes into one or more requirements and grouped them into five different domains [68]: (1) functional and modality requirements, specifying technical features and prescribing the type of technology and operating systems; (2) content requirements, specifying the content to be communicated via the technology; (3) user experience requirements, specifying how the technology should interact and communicate with the user; (4) service requirements, specifying desired services surrounding the technology (ie, user support); and (5) work context requirements, specifying how the technology should be integrated into the existing rehabilitation context and routines.

Credibility of the findings was enhanced through regular peer debriefing sessions held every 2 weeks with experts from the quality assurance consultants of the largest statutory accident insurer in Germany. These external experts provided feedback on our findings, helping to validate and refine the identified themes and ensuring their accuracy and relevance.

The sample size in this study was based on the concept of theoretical saturation [69], which is defined as the point at which no new information, themes, or topics emerge from the data. Saturation in this context means that no new subthemes of facilitators or subthemes of barriers or attributes could be identified. The number of codes (subthemes and requirements) was summarized descriptively for all 27 participants and separately for the 2 subgroups of patients and personnel.

### Ethical Considerations

The study was conducted in accordance with the Code of Ethics of the Declaration of Helsinki and was reviewed by the local psychological ethics committee at the Center for Psychosocial Medicine (Hamburg, Germany; process number LPEK-0248). We obtained written informed consent from all participants before their participation. All data were anonymized after the interviews were transcribed. The participants did not receive any compensation for their participation in the study.

## Results

### Key Stakeholders

**Figure 1:**
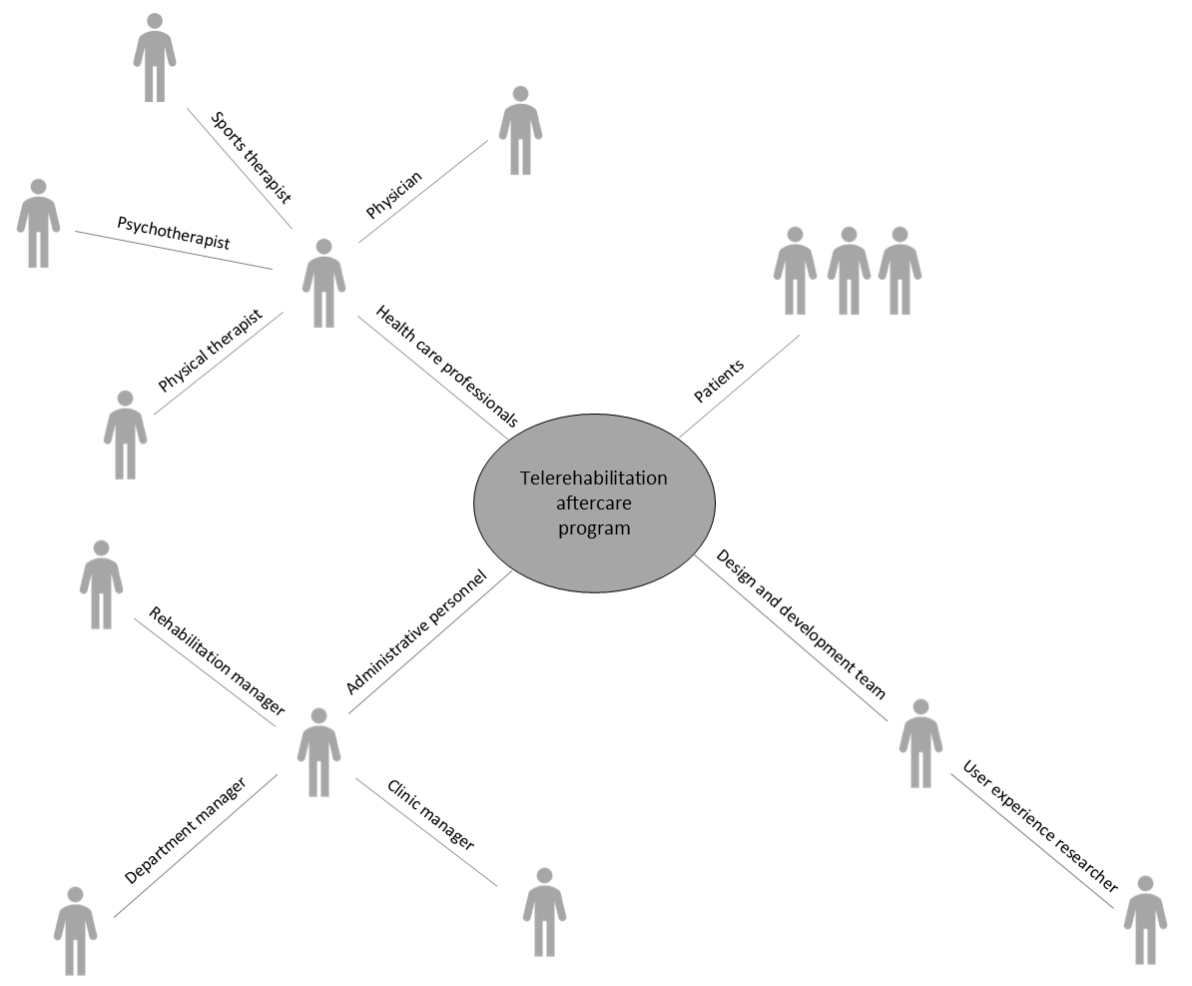
Key stakeholder map.

The expert group identified key stakeholders, categorizing them into four groups: (1) patients after occupational accidents who require rehabilitation, (2) health care professionals who treat patients after occupational injuries, (3) administrative personnel, and (4) members of the design and development team of telerehabilitation programs. The final map of key stakeholders is displayed in Figure 1.

### Participants’ Characteristics

Participant characteristics are presented separately for the patients (Table 1) and the personnel, including health care professionals, administrative personnel, and design and development team members (Table 2).

**Table 1:** Patients’ sociodemographic and medical characteristics and internet use (N=7).

Patients’ characteristics		Values
Age (years), mean (SD; range)		48.9 (14.1; 31-77)
Sex (female), n (%)		2 (29)
**Profession, n (%)**		
	Bus driver	1 (14)
	Secretary	1 (14)
	Janitor	1 (14)
	Supervisor of gambling halls	1 (14)
	Technical director of a church	1 (14)
	Underground construction manager	1 (14)
	Trainer of horses and riders	1 (14)
Patient uses the internet, n (%)		6 (86)
Patient has prior experience with digital health care services (yes), n (%)		2 (29)
Average time spent on the internet per day (hours), mean (SD; range)		1.5 (1.5; 0-4)
**Preferred device for internet access, n (%)**		
	Smartphone	4 (57)
	Tablet	2 (29)
	None	1 (14)
**Care setting the patient was treated in, n (%)**		
	Outpatient treatment	4 (57)
	Inpatient and outpatient treatment	3 (43)
Time since injury (months), mean (SD; range)		12.9 (15.3; 1-36)
**Type of injury (*ICD-10*^a^ code), n (%)**		
	Biceps tear (S46.2)	1 (14)
	Fracture of lateral malleolus (S82.6)	1 (14)
	Dislocated shoulder (S43.0)	1 (14)
	Fracture of the lumbar vertebra (S32.0)	1 (14)
	Patellar tendon rupture (S76.1)	1 (14)
	Fracture of the tibial spine (S82.11)	1 (14)
	Fracture of the leg in 12 different places (not defined)	1 (14)

^a^ICD-10: International Classification of Diseases, Tenth Revision.

**Table 2:** Personnel sociodemographic and work-related characteristics (N=20).

Personnel’s characteristics		Values
Age (years), mean (SD; range)		40.9 (11.5; 27-63)
Sex (female), n (%)		10 (50)
**Profession, n (%)**		
	Physical therapist	4 (20)
	Rehabilitation manager	3 (15)
	Medical director of a rehabilitation clinic	2 (10)
	Psychotherapist	2 (10)
	Sports therapist	2 (10)
	Physician	1 (5)
	Clinic manager for physical and massage therapy	1 (5)
	Clinic site managers	1 (5)
	Department manager of rehabilitation (organizational and personnel management)	1 (5)
	Clinic manager for prevention and aftercare	1 (5)
	Clinic manager for physical, sports, and occupational therapy	1 (5)
	User experience researcher	1 (5)
**Work setting, n (%)**		
	Outpatient treatment	8 (40)
	Administrative setting	6 (30)
	Administrative setting and outpatient treatment	3 (15)
	Inpatient and outpatient treatment	2 (10)
	Research setting	1 (5)
**Work experience (years)**		
	Mean (SD; range)	11.5 (8.0; 1-25)
	Missing values, n (%)	3 (15)
Prior work experience with telerehabilitation, n (%)		9 (45)

The age of the patients ranged from 31 to 77 years (mean 48.9, SD 14.1 years). Almost three-quarters (5/7, 71%) of the patients were male. All patients had different occupations. Of the 7 patients, 1 (14%) did not use the internet and 2 (29%) had prior experience with digital health care services (eg, digital fitness or mental health applications). The patients spent an average of 1.5 (SD 1.5) hours per day on the internet. The most preferred device for internet access was the smartphone (4/7, 57%). Most patients (4/7, 57%) were treated in an outpatient setting. The mean time since the occupational injury was 12.9 (SD 15.3) months. Of the 7 patients, 2 (29%) were in their third year of treatment, while 4 (57%) were in their first 6 months.

The personnel’s age ranged from 27 to 63 years (mean 40.4, SD 11.5 years; Table 2). An equal number (10/20, 50%) of male and female personnel participated. The largest professional groups were physiotherapists (4/20, 20%) and rehabilitation managers (3/20, 15%). Almost half of the personnel (8/20, 40%) worked in an outpatient setting. Their mean work experience was 11.5 (SD 8.0) years. About half of the personnel (9/20, 45%) had prior experience with telerehabilitation programs. The personnel worked in 11 different clinics and institutions. The largest group of 1 clinic consisted of 5 participants working in the same outpatient rehabilitation clinic.

### Number of Identified Codes and Theoretical Saturation

A total of 91 codes (subthemes and attributes) were identified across all 27 transcripts, and they included 27 subthemes of facilitators, 38 subthemes of barriers, and 27 requirements. In total, 33 codes were identified in the patient subgroup, all of which were already identified by the first 6 (86%) of the 7 participants. In the personnel subgroup, we identified 87 codes, 86 (99%) of which were identified by the first 19 (95%) of the 20 participants.

### Facilitators and Barriers to a Telerehabilitation Aftercare Program

#### Overview of Facilitators and Barriers

The barriers and facilitators of the prospective telerehabilitation program for patients after occupational accidents are described in the 3 subsections: individual, technical, and environmental and organizational facilitators and barriers. The most frequently reported overarching themes for facilitators were time savings, patient motivation, high usability, and regular in-person therapy meetings during the telerehabilitation aftercare program (Multimedia Appendix 3). The most frequently reported overarching themes for barriers were low digital affinity and skills, lack of trust and acceptance of telerehabilitation programs, fear of less social interaction through the telerehabilitation aftercare program, slow internet speed, program functionality problems (eg, application crashes and freezes) of telerehabilitation programs, and that not all therapy elements can be executed over the internet (Multimedia Appendix 4).

#### Individual Facilitators

The key stakeholders reported time savings for patients, personnel’s motivation, patient motivation, and the availability of necessary hardware for patients as individual facilitators of the telerehabilitation aftercare program. Patients were expected to save time because telerehabilitation eliminates travel time, and the telerehabilitation program could be flexibly integrated into daily routines:

Because they [patients] like the fact that they can do it flexibly from home. And then they don’t have to go back and forth all the time.

User experience researcher

The personnel indicated that their motivation would increase if they were involved in the telerehabilitation program development process, were interested in the patient’s well-being, and trusted the telerehabilitation aftercare program. The personnel expected that patient motivation would be higher for patients with high self-motivation, patients who set and achieve rehabilitation goals, patients who have high acceptance of the telerehabilitation program, and patients who receive support from their family. In addition, they experienced that some patients felt more motivated in a group exercise program than in an individual exercise program.

Moreover, the availability of the necessary hardware (eg, tablet, smartphone, or computer) for patients was identified as an individual facilitator.

#### Technical Facilitators

Key stakeholders reported high program usability, data privacy protection, high program functionality, and web anonymity as technical facilitators of the telerehabilitation aftercare program. For the program’s usability, it is essential that access is easy, the design is clear, and the interface is user-friendly:

For me, it’s very important, and this is actually one of the most important things, that an app like this is, let’s say, very easy to use, both for the user, the patient, and for the therapist. Because the therapists, if they have to be instructed in a very complex way, it is very time-consuming, which is now quite difficult.

Clinic manager for physical, sports, and occupational therapy

The personnel expected that patients would be more likely to participate in the telerehabilitation aftercare program if they felt that their data were secure and the program had a consistent functionality. In addition, personnel indicated that the anonymity of digital programs could help patients who have prejudices against psychotherapy and fear stigmatization to make initial contact.

#### Environmental and Organizational Facilitators

The key stakeholders identified having regular in-person therapy meetings during the telerehabilitation aftercare program, therapists’ work schedule with fixed time slots for the telerehabilitation aftercare program, patients receiving in-person rehabilitation and the telerehabilitation aftercare program from the same therapist, well-equipped therapy rooms, financial support for patients to purchase necessary hardware, and cost savings as the environmental and organizational facilitators of the telerehabilitation aftercare program. The personnel demanded that therapists should get fixed time slots where they can do telerehabilitation work. The personnel suggested that having the same therapist for in-person rehabilitation and the telerehabilitation aftercare program would increase the quality of therapy because therapists would be more likely to adapt the exercise program to the patients’ needs. The personnel reported that rehabilitation clinics would need therapy rooms or stations with hardware, exercise equipment, and a stable Wi-Fi connection for health care professionals to interact with patients. The personnel who reported this facilitator envisioned a real-time format of the telerehabilitation program, with patients and therapists connected via a video call. The personnel also noted that patients without access to the necessary hardware for the telerehabilitation aftercare program would require some form of financial assistance. In addition, the key stakeholders expected that the telerehabilitation aftercare program would save costs compared to in-person rehabilitation because, for example, fewer therapists would be needed if patients received their exercise instructions via video:

In addition, you also save money as a company. Of course, as a company, you have to invest first, no question about that, but you also ultimately save costs that still incurred here in the house, because in principle you then only have one therapist, maybe two, who ultimately go into the exchange with the patient.

Sports therapist

#### Individual Barriers

The key stakeholders reported low digital affinity and skills, low language and cognitive skills of the patients, patients being unable to perform exercises without assistance, patients not having the necessary hardware, patients’ home not suitable for telerehabilitation, lack of trust and acceptance, fear of less social interaction, low patient motivation, personnel’s concern of being replaced, and exercises that are too difficult or not related to work activities as individual barriers to telerehabilitation aftercare program. The key stakeholders indicated that low digital affinity and skills of the patients and personnel would be a barrier to the telerehabilitation aftercare program. They also experienced that older age is negatively associated with digital skills:

It also depends on the patient clientele, the younger ones are sometimes a bit more open to it and the older generation less so, of course they already have these problems with the technology.

Patient

The personnel stated that patients with poor German language skills and cognitive impairments (ie, after an accidental brain injury) would not be able to participate in the telerehabilitation aftercare program. Similarly, patients who are unable to perform the therapy exercises on their own would still need in-person therapy. The personnel pointed out that patients often do not have the privacy they need at home, for example, to discuss intimate issues with a psychotherapist or to exercise without interruptions. The key stakeholders believed that patient and personnel acceptance of a telerehabilitation aftercare program would be low and that few patients would participate. The key stakeholders reported that patients and health care professionals would miss in-person interactions and that it would be easier to establish a therapeutic relationship to discuss the sensitive issues in an in-person setting. They also expected that patient adherence would decrease due to less social interaction in telerehabilitation compared to in-person rehabilitation. In addition, the personnel identified low patient motivation as an individual barrier. They expected that patients with low motivation would not be able to complete the full telerehabilitation aftercare program. The personnel reported that physical therapists were concerned that their work would be replaced by telerehabilitation. In addition, the key stakeholders described previous experiences with video-instructed exercises that were too difficult for patients recovering from accidental injuries or not relevant to their work activities:

Like I said, and with a telerehabilitation program, we’re still missing a little bit of the working life reference. They have a few exercises on lifting and carrying, but that’s the best they can do.

Clinic manager for prevention and aftercare

#### Technical Barriers

The key stakeholders identified slow internet speed, lack of data protection, privacy concerns or issues, functionality problems of the telerehabilitation program, the fact that not all therapy elements can be done over the internet, and different digital programs for different health care providers as technical barriers to the telerehabilitation aftercare program. Slow internet speed in rehabilitation clinics and patients’ homes were expected to reduce patient participation in the telerehabilitation aftercare program:

Then, as we can see right now, the Internet connection is also critical for us. The Wi-Fi connection can be very poor or very irregular.

Physical therapist

The key stakeholders reported patient and personnel concerns about privacy, which could negatively impact personnel and patient acceptance of the program. They also believed that functionality problems (eg, application crashes and freezes or log-in and authentication issues) of the telerehabilitation program would lead to user frustration. The key stakeholders expected that certain elements of rehabilitation for patients after occupational injuries could not be performed via digital programs. For example, in a telerehabilitation setting, physical therapists would not be able to monitor whether and how patients were performing exercises, which could lead to patients performing exercises incorrectly and increasing their risk of reinjury. They pointed out that haptic therapy elements, such as mechanotherapy, require physical examination and cannot be delivered via digital programs. In addition, 2 psychotherapists stated that they could not perform exposure therapy via digital programs. The clinic manager for prevention and aftercare experienced that, for technical reasons, it is not possible to tailor the exercises to the needs of the patients. One patient reported that he would not use telerehabilitation if different health care providers, such as his health insurance and the statutory accident insurance, used different mobile apps.

#### Environmental and Organizational Barriers

The key stakeholders identified high costs and implementation issues as environmental and organizational barriers to the telerehabilitation aftercare program. They expected high costs for a Wi-Fi expansion in the rehabilitation clinics and high costs for the telerehabilitation provider. They assumed that there would be a risk of providers going bankrupt because telerehabilitation is a new field and no one knows which of the companies will prevail. The anticipated implementation problems were not meeting the implementation time schedule and changing the decision makers’ opinion about the telerehabilitation aftercare program during the implementation process. It was expected that not meeting the implementation time schedule would decrease the motivation of health care professionals to participate in the telerehabilitation aftercare program because they would lose interest in it:

If we start a project like this now, or if we say here in the administration: “We want to tackle this now,” and then it takes another year and a half to actually implement it, then the willingness to do it, or to participate in it, or the interest in it, simply fades.

Department manager of rehabilitation

### Design Requirements for a Future Telerehabilitation Aftercare Program

#### Overview of Design Requirements

Separate descriptions for the content, function, service, user experience, and working context requirements, including sample citations to illustrate the underlying data, are provided in Multimedia Appendix 5 [68]. The requirements mainly center around the identified barriers and facilitators. The 3 most frequently discussed overarching values were tailored intervention, social interaction, and integration in therapists’ work context.

#### Content Requirements

The key stakeholders requested that the telerehabilitation aftercare program should include tailored exercise plans with exercise videos. The videos should feature audiovisual instructions on how to perform the tailored exercises, common difficulties in performing the exercises, and common evasive movements. Exercise plans should be tailored to the patients’ type of occupational injury, functional status, goals, and job requirements. For example, patients with desk jobs will need different exercises than patients who do heavy physical labor:

In any case, that you are shown the exercise once. What difficulties there might be or what to look for. What evasive movements are common. How to counteract them.

Physical therapist

The key stakeholders would be interested in a combination of digital and in-person rehabilitation aftercare, as in a blended care approach. Patients would receive a combination of telerehabilitation with regular in-person therapy sessions (eg, every 2 weeks). The regular face-to-face therapy sessions would allow, for example, monitoring of exercise performance. The key stakeholders would like patients to get access to tailored information on how to cope with their injury in everyday life and nutritional information. The information should help answer questions such as “Does the patient need to change his or her living situation or job, such as living in a house without stairs or working a desk job because heavy physical labor is no longer possible?” or “Do people need to change their diet to optimize the rehabilitation process?” In addition, the key stakeholders wanted patients to receive psychotherapy via a real-time video call with a psychotherapist, a selection of relaxation exercises for patients to choose from, and the ability to access their medical records.

#### Functional Requirements

The key stakeholders wanted the main component of the telerehabilitation aftercare program to be an web-based platform that patients can access to find videos and written information that includes all the content described in the content requirements section. The program should be easily accessible from a computer or a mobile device. If the program would be available as a mobile app, it should be available for iOS and Android and should also work on older hardware:

So it would work on mobile devices like tablets or cell phones or smartphones or even computers? Especially for older people or if the phone is too old or something.

Physical therapist

The key stakeholders wanted the telerehabilitation aftercare program to enable communication through real-time video calls among patients, health care providers, and rehabilitation managers. Hereby, patients can participate in individual and group exercise therapy, psychotherapy, and medical consultations (with their physician). Physical therapists can guide, observe, and correct patients as they perform exercises. In addition, a psychotherapist was interested in being able to share his screen during the video call to show his notes to the patient.

The key stakeholders also requested that the exercise plans be easy to create and adjust. Health care professionals should be able to choose from predesigned exercise plans. The predesigned exercise plans should vary based on the patients’ type of occupational injury, functional status, goals, and job requirements. Selecting a predesigned exercise plan and tailoring it would save time compared to compiling exercise videos from a large pool of videos that meet the patients’ needs. In addition, the key stakeholders wanted therapists to be able to change the frequency and intensity of exercises and repeat exercise plans with a single adjustment. In addition, the personnel requested that patients should have the option to adjust the intensity of the exercise within a range predefined by the therapist. For example, if the patient was in good daily form, they could change the number of repetitions of an exercise from 8 to 10.

The key stakeholders requested an asynchronous communication feature that would allow patients and health care professionals to communicate with each other. The patient could use a chat function or a voice message to comment on his or her exercise session, to share photos of swollen limbs with his or her doctor, or to contact his or her rehabilitation manager about problems at work or in the social context. Health care professionals should receive an overview of all the messages received when they log into the program and could then respond. The personnel suggested that patients’ exercise execution could be evaluated through motion tracking. For example, is there sufficient range for wrist flexion and extension? Patients would receive real-time feedback on their execution and could make adjustments as needed.

In addition, the personnel would like therapists to be able to record and share their own exercise videos, tailored to the needs of an individual patient. These videos could be important if the video platform does not include videos related to the patients’ specific job requirements.

The personnel suggested that the program should provide daily reminders for patients to execute their exercises. The reminders should be sent via email or as a push notification on the smartphone. They also requested that the program should be available in different languages. Patients, especially in the construction industry, who are temporary workers from other European countries would benefit from a telerehabilitation aftercare program in their native language.

The key stakeholders wanted patients to be able to submit applications and track the status of their claims and decisions related to the statutory accident insurance scheme. They wanted to have all correspondence and information related to the accidental injury available in 1 place.

In addition, the personnel requested that the program should comply with the European Unions’ General Data Protection Regulation.

#### Service Requirements

The personnel expressed a desire to have a contact person at the telerehabilitation program provider whom they could approach with suggestions for improving the telerehabilitation aftercare program. Suggestions should be discussed and the program adjusted if necessary:

Anyhow, that you have a good contact person, that you can reach 24/7.

Clinic manager for prevention and aftercare

The personnel requested that they receive recurring training from the telerehabilitation provider’s staff on how to use the telerehabilitation aftercare program. The user experience researcher noted that therapists often use only a few settings to modify patients’ exercise plans, which she believed was because they have not been instructed on how to use other settings. At the end of the rehabilitation, patients should receive a content and technical introduction to the aftercare program from trained therapists. In this way, initial difficulties with the program could be resolved in an in-person context to increase the acceptance of the program. The key stakeholders requested that the telerehabilitation provider establish a technical support hotline for personnel and patients to call in case of technical issues. Furthermore, a psychotherapist suggested that patients who do not have the necessary hardware to participate in the telerehabilitation aftercare program should receive financial assistance to purchase the hardware.

#### User Experience Requirements

The key stakeholders required high usability of the telerehabilitation aftercare program. The program should be clear and self-explanatory for the patients and personnel. Font sizes and contrast levels would be adjusted to ensure they are suitable for older patients. The key stakeholders anticipated that the biggest challenge would be finding a balance between providing users with functional options and avoiding an overload of choices. The user experience researcher indicated that a possible solution to this challenge would be for the program to guide users step-by-step through a predefined sequence of actions called tunneling. Tunneling removes any unnecessary features that might distract the user from completing the process:

So that the system guides you step by step through the individual tasks that I need to complete.

User experience researcher

In addition, the personnel would like to see patients receive written praise from the program for completing exercises, for example, “You have done your exercises for seven days, great job!”

#### Work Context Requirements

The personnel requested a digital workstation or offices for health care professionals. The offices should be equipped with advanced computer or mobile devices and a fast internet connection. Physical therapists would need high-definition cameras, enough space, and exercise equipment to demonstrate exercises in real-time video calls:

The prerequisite, of course, is that you have space, not just a PC, but maybe also a mat where you can actively show and demonstrate certain exercises. That means, of course, that the room needs to be a certain size.

Physical therapist

In addition, the personnel requested that health care professionals be given set periods during which they can perform telerehabilitation tasks, for example, to read patient comments about their exercises, to provide feedback to patients, or to create tailored exercise plans. The personnel expressed concern that these tasks could be scheduled as overtime.

The personnel requested that the same therapist guide patients through in-person rehabilitation, train them to use the telerehabilitation program toward the end of in-person rehabilitation, and support patients in telerehabilitation. They indicated that to support patients well in the telerehabilitation aftercare program, it is essential that they have already established a relationship during the in-person rehabilitation.

## Discussion

### Principal Findings

The overall goal of this study was to explore the facilitators of and barriers to a future telerehabilitation aftercare program for patients after occupational injuries from the perspective of key stakeholders and then translate these perspectives into concrete design requirements that can guide the development of a future telerehabilitation aftercare program.

The most frequently reported facilitators were time savings for patients, as telerehabilitation would eliminate travel time; high patient motivation to participate in the telerehabilitation aftercare program; high usability of the program; and having regular in-person therapy meetings during the telerehabilitation aftercare program. The most frequently reported barriers were low digital affinity and skills of the patients and personnel; lack of patient trust and acceptance of the telerehabilitation aftercare program; less social interaction, which could reduce therapy adherence and make it more difficult to establish and maintain a therapeutic relationship; slow internet speed in rehabilitation clinics and in patients’ homes; problems in the functionality of the telerehabilitation program; and the inability of the telerehabilitation program to perform certain elements of in-person rehabilitation aftercare, such as monitoring exercise execution, conducting physical examination, and providing haptic feedback.

Content, functional, service, user experience, and work context requirements for a telerehabilitation aftercare program were translated from key stakeholder statements. The 2 most frequently discussed overarching values were program tailoring and social interaction. Tailoring telerehabilitation content to the patients’ type of occupational injury, functional status, goals, and work requirements, such as tailored exercise plans, tailored injury-related information, and a selection of relaxation exercises, was a key value for key stakeholders to ensure that the program could meet the needs of patients with different types of occupational injuries. Key stakeholders believed that tailoring would increase the effectiveness of the program and improve patient adherence. Requirements such as real-time psychotherapy, digital and in-person rehabilitation aftercare in a blended care approach, real-time video calls, dialogue support such as praise [70] for completing exercises, and having the same therapist for in-person rehabilitation and the telerehabilitation aftercare program could reduce concerns that telerehabilitation would decrease social interaction between the patients and therapists. Key stakeholders felt that in a digital rehabilitation format, social interaction is important for establishing and maintaining a therapeutic relationship, for regular monitoring of exercise performance, and for fostering therapy adherence.

### Context and Comparison With Previous Work

Our findings regarding the most frequently reported barriers and facilitators are consistent with the results of previous systematic reviews that analyzed barriers and facilitators of synchronous telerehabilitation programs for patients with musculoskeletal disorders [53], real-time web-based consultations for patients with various health conditions [52], and eHealth programs in general [51]. However, in contrast to the earlier research [51-53], key stakeholders in our study were more concerned that certain elements of in-person rehabilitation could not be performed digitally, especially the monitoring of exercise performance. This may be explained by the fact that almost half of the personnel (9/20, 45%) had previous experience with 2 telerehabilitation programs that primarily use exercise instruction videos. In the video instruction format, therapists cannot monitor and control the execution of the exercise as they can in synchronous telerehabilitation formats such as real-time video calls and blended care approaches [53]. Participants also did not appear to be aware of programs or digital applications that provide real-time feedback (eg, Kinect sensor for motion tracking) on exercise execution. Recent study results indicate the effectiveness of these types of programs [29,30,40,44].

The design requirements in our study were mostly elicited to reduce barriers and realize facilitators. For example, service requirements such as technical support hotlines for personnel and patients, recurrent training of personnel on how to use the telerehabilitation aftercare program by personnel of the telerehabilitation provider, and patients being introduced to the telerehabilitation aftercare program during rehabilitation could be encouraging for the patients and personnel with low digital affinity and skills and lower barriers such as low trust and low acceptance of telerehabilitation programs and functionality problems of telerehabilitation programs.

However, for 3 barriers, no requirements could be identified to reduce the barrier. If the patients do not have the privacy at home to discuss intimate issues with a psychotherapist or to exercise without interruption, if they have low cognitive abilities, or if the internet connection is too slow, they would simply not be able to participate in the telerehabilitation aftercare program. In addition, providing financial support or loaning hardware to the patients who do not have the necessary hardware was not well received by the quality assurance consultants at the accident insurance provider.

To reduce some barriers, >1 potential design requirement was identified. As mentioned in the Context and Comparison With Previous Work section, regular monitoring of the patients’ exercise performance was an important theme in many interviews. Key stakeholders felt that exercise performance should be monitored regularly to prevent patients from performing exercises incorrectly [28] and even reinjuring themselves. Key stakeholders presented 3 different possible approaches to this issue, which have been successfully used in previous telerehabilitation studies for patients with orthopedic injuries and disorders. First, 15 key stakeholders discussed the possibility of real-time video calls using high-definition cameras. Physical therapy via real-time video calls has been effectively used as an exclusive treatment modality for patients after TKA [46,47] or as a regular monitoring element to supplement exercise instructions via video [32,45] for patients after total hip arthroplasty [32] and for patients with proximal humerus fractures [45]. Second, 6 key stakeholders discussed a blended care approach of telerehabilitation and in-person rehabilitation aftercare, which has also been used effectively for patients after total hip arthroplasty, TKA [28], or flexor digitorum profundus tendon repair [33]. Four key stakeholders discussed the possibility of motion tracking with real-time feedback to the patients. Motion tracking is a form of external feedback that can contribute to the learning of a new motor skill, as in rehabilitation [71]. It would also be possible to combine the approaches, such as the combination of motion tracking and video calls for patients after TKA [30]. We would expect the real-time video method to be the most expensive, as it requires the most time and effort from the therapists. To complete the value specification phase of the telerehabilitation aftercare program development, 2 focus groups should be held to reach consensus on the design requirements of the telerehabilitation aftercare program [57,72,73]. In the 2 focus groups, a low-fidelity prototype (ie, a sketch or storyboard), including the functional and user experience requirements, should be used to facilitate the requirements specification process, as stakeholders often have difficulty identifying attributes and requirements if they do not know what the technology might come to look like [72,73]. Subsequently, the iterative phases of the CeHRes roadmap [56,57,60] should be followed to develop a user-friendly telerehabilitation aftercare program for patients with occupational injuries.

### Limitations

This study had some limitations. First, it was difficult for key stakeholders to talk about barriers, facilitators, and especially potential features and requirements of a telerehabilitation aftercare program due to their lack of experience or expertise in the specific topic. This was especially true for patients, as only about one-third of the patients (2/7, 29%) had previous experience with digital health services, and 1 (%) patient did not use the internet at all. Among personnel, the problem was less severe because they were recruited only from clinics that already used telerehabilitation programs, and almost half of the personnel (9/20, 45%) had prior work experience with telerehabilitation programs. The patients made an average of 12.6 (SD 7.6) codable expressions per interview, while the personnel made 27.3 (SD 7.6). The best example of this challenge was the user experience requirement for high usability of the telerehabilitation aftercare program. A total of 14 key stakeholders recommended that the program should be self-explanatory and easy to use, but only the participating user experience researcher was able to articulate specific requirements such as using font sizes suitable for older adults or implementing tunneling techniques. It would have been a good idea to use existing or potential examples of telerehabilitation programs, such as low-fidelity prototypes, to support patient interviews [65,72,73]. It might have been easier for the patients to formulate their needs using concrete examples, “instead of coming up with it out of thin air” [73].

Second, we did not involve stakeholders in the determination of the key stakeholders. We used expert discussion alone instead of involving stakeholders in this discussion [60,68] or a combination of expert discussion and stakeholder involvement methods, such as the one proposed by Mitchell et al [74], which has been used in previous similar studies [72,75]. Therefore, we cannot completely exclude the possibility that we might have overlooked an important key stakeholder or overestimated the importance of the views of certain stakeholder groups for the design and implementation process. An important stakeholder group that we might have overlooked is the family or relative group. Family support has been identified as a facilitator of exercise-based rehabilitation in this study and in previous studies [76-79], and interviews with family members or caregivers might have led to the identification of additional barriers, facilitators, or requirements. Third, using the concept of theoretical saturation [69] also captures the risk of missing additional barriers, facilitators, attributes and requirements [80]. In particular, the small number of patients who participated in the study might be of concern, given the wide variety of different occupational injuries [3,4]. As mentioned in the first limitation, the patients had difficulty in reporting barriers, facilitators, and requirements because they had less experience with rehabilitation or telerehabilitation than the personnel. Distracting screens, patient privacy concerns, and lack of exercise equipment were the only codes reported only by the patients and not by the personnel. However, we consider that interviewing more patients for the study would not have resulted in finding additional important barriers, facilitators, attributes or requirements that were not already expressed by the participating patients and especially by the more experienced personnel, as no new codes were identified in the seventh patient transcript.

Fourth, the identification of attributes and requirements might have been subjective on the part of the researchers. Statements such as “these are the relaxation exercises that we offer and you just choose them yourself” made by the key stakeholders left little room for interpretation. Other statements such as “via this platform the patient can also get in touch with the doctors and therapists” left more room for interpretation. Nevertheless, to avoid subjective interpretation, 19% (5/27) of the interviews were coded by a second researcher. Differences in codes schemes were discussed and resolved between the 2 researchers.

### Conclusions

Carefully conducted contextual inquiry and value specification with the involvement of key stakeholders is a crucial step in the development of a telerehabilitation aftercare program. The key stakeholders identified facilitators, barriers, and design requirements that should be accounted for throughout the development process. Tailoring of telerehabilitation content, such as tailored exercise plans, tailored injury-related information, and a selection of relaxation exercises, was a key value for stakeholders to ensure that the program could meet the needs of patients with different types of occupational injuries. An important decision that remains to be made after further focus groups with key stakeholders is the selection of an approach for exercise monitoring.

## Appendix

Multimedia Appendix 1 Standards for Reporting Qualitative Research.

Multimedia Appendix 2 Interview topic guides.

Multimedia Appendix 3 Expected facilitators of the telerehabilitation aftercare program for patients after occupational injuries.

Multimedia Appendix 4 Expected barriers of the telerehabilitation aftercare program for patients after occupational injuries.

Multimedia Appendix 5 Requirements of the telerehabilitation aftercare program for patients after an occupational accident.

## Abbreviations

CeHRes: Center for e-Health Research and Disease Management
TKA: total knee arthroplasty

## Data Availability

The datasets generated and analyzed during this study are available from the corresponding author upon reasonable request.
