# Supplementary material for: Barriers, Facilitators, and Requirements for a Telerehabilitation Aftercare Program for Patients After Occupational Injuries: Semistructured Interviews With Key Stakeholders
Source: JMIR Form Res. 2024 Nov 8;8:e51865. doi: 10.2196/51865 (PMC11584548; doi:10.2196/51865)
Supplement: Multimedia Appendix 2 [file formative_v8i1e51865_app2.docx]

Multimedia Appendix 2: Interview topic guides
Interview topic guide used with patients with occupational injuries

| Topic | Question/Instruction |
| --- | --- |
| Introduction | - Introduction of the researcher and presentation of the study objective  - Content, structure, and duration of interview  - No right or wrong answers; participants can skip questions they do not want to answer  - Participant can ask questions at any time  - Does the participant have any questions so far? |
| Socio-demographic and medical characteristics/ internet use | - What is your age?  - What do you do for a living?  - What kind of occupational injury did you have?  - How much time has passed since the occupational accident?  - Do you use the Internet/digital services?  - How much time do you spend online on average every day?  - What device (computer, smartphone, etc.) do you use to access the internet? |
| Grand tour question/ facilitators of a telerehabilitation aftercare program | - What has been your experience with digital health care services so far?  **Probes:** What positive experiences have you had with the services? What could be improved about the service you used (technical, content)?  - What if the patient has no prior experience? What prevents you from using digital services? (Barriers) |
| Patients' needs/current situation of rehabilitation after occupational accidents | - What are special challenges after an accidental injury?  - What are strong and weak points of the current rehabilitation program (for patients after occupational accidents)?  - Would you like to continue your rehabilitation after you return to work?  - What might be the content of such an aftercare program?- Do you/other patients need support regarding emotional distress?  - Do you/other patients need more information regarding treatment of your/their occupational injury?  - What are potential barriers to participation in a rehabilitation aftercare program?  What do you think could possibly prevent other patients with an accident injury from participating in a rehabilitation aftercare program? |
| Facilitators/Requirements of/for a telerehabilitation aftercare program | - Do you prefer a digital or analog rehabilitation aftercare program?  - What do you think are the benefits of a telerehabilitation aftercare program?  - What do you think are the advantages of an analog rehabilitation aftercare program?  - What features would a digital telerehabilitation aftercare program need in order for you to participate?  Probes: What content would you like to access online?  What online feedback would you need from your healthcare provider?  - How would you feel about a blended digital and analog rehabilitation aftercare program? |
| Barriers of a telerehabilitation aftercare program | - What are possible barriers to participating in a telerehabilitation aftercare program?  What do you think might prevent you or other TBI patients from participating in a telerehabilitation follow-up program? |
| Ending the interview | - Is there anything else I've forgotten that I haven't asked you about but you think is important in this context? Is there anything else you would like to add? |

Interview topic guide used with health care professionals and the human factor expert

| Topic | Question/Instruction |
| --- | --- |
| Introduction | - Introduction of the interviewer and presentation of the study objective  - Content, structure, and duration of interview  - No right or wrong answers  - Participants can skip questions they  prefer to not answer  - Participant can ask questions at any time  - Do the participants have any questions so far? |
| Socio-demographic and work-related characteristics | - What is your age?  - What is your job title?  - Can you briefly describe your responsibilities in this job?  - How many years have you been providing patient care?  - Do you work with patients covered by the statutory accident insurance? |
| Grand tour question/ requirements for a telerehabilitation aftercare program | - In your opinion, what should the perfect telerehabilitation aftercare program for patients after occupational injury look like?  - What has been your experience with digital health care services so far? |
| Current situation of telerehabilitation programs | -What are problems of current telerehabilitation programs from your point of view?  -If the participant had no prior experience? Skip the question |
| Introduction of individual, environmental and organizational, and technical facilitators and barriers | First, individual barriers and facilitators are characteristics or attributes of subsequent users of the telerehabilitation program, i.e., primarily patients, but also health care professionals (e.g., physicians, physical therapists, etc.), that prevent or facilitate the use of a telerehabilitation service.  Second, environmental and organizational barriers and facilitators include, for example, the requirements and structures of rehabilitation clinics or incentives for physicians or clinics to offer telerehabiliation programs to their patients.  Technical barriers and facilitators are reflected in issues such as ease of use, standards for patient-generated data, or technical support when problems occur. |
| Individual Facilitators and barriers/Added value of a telerehabilitation aftercare program | - What are important individual facilitators of a telerehabilitation aftercare program for patients with accidental injuries?  - What are important individual barriers to participation in a telerehabilitatin aftercare program for patients with accidental injury?  Probes: What special characteristics do patients with accidental injuries bring to the table? What needs do they have? What current issues are the patients facing? What is important to you as a physician/therapist when caring for patients with accidental injuries? What characteristics should a telerehabilitation aftercare program have so that you would participate? |
| Environmental and organizational barriers and facilitators | What environmental and organizational requirements have to be addressed so that you would be able to offer/support a telerehabilitation aftercare program at your clinic?  Probes: What personnel/financial requirements would have to be fulfilled for successful implementation? What are current structural and organizational barriers at your clinic? |
| Technical barriers and facilitators/ requirements for a telerehabilitation aftercare program | What are the technical requirements for a telerehabilitation program to be successfully implemented for patients after occupational accidents?  Probes: What technical features/functions have patients and/or health care professionals had problems with in digital products you may be familiar with? What technical features are important to you? |
| Ending the interview | - Is there anything else I've forgotten that I haven't asked you about but you think is important in this context? Is there anything else you would like to add? |
