# Supplementary material for: Barriers, Facilitators, and Requirements for a Telerehabilitation Aftercare Program for Patients After Occupational Injuries: Semistructured Interviews With Key Stakeholders
Source: JMIR Form Res. 2024 Nov 8;8:e51865. doi: 10.2196/51865 (PMC11584548; doi:10.2196/51865)
Supplement: Multimedia Appendix 3 [file formative_v8i1e51865_app3.docx]

**Multimedia Appendix 3.** Expected facilitators of the telerehabilitation aftercare program for patients after occupational injuries.

| Type of facilitator | Theme | Subtheme | Example quotes | Stated by | | Number of Stakeholders^a^ |
| --- | --- | --- | --- | --- | --- | --- |
|  |  |  |  | Personnel | Patients |  |
| Individual | Saving time | Reducing travel time for patients | *Because they are happy about the fact that they can do it flexibly from home. And then they don't have to go back and forth all the time* | X | X | 11 |
|  |  | Flexibility in daily routines for patients | *Or also X, this, how do you say, if you don't want to have this commitment now, for example, or appointments, that you are a bit more flexible. I think for patients like that, it's quite a good thing.* | X | X | 6 |
|  | Patients’ motivation | High Self-motivation | *The premise or characteristic is simply that they can manage without me as a therapist.* | X |  | 7 |
|  |  | Set goals/experience moments of success | *So, they often need external motivation to do their things. And perhaps also a perspective or a goal. In therapy, we also try to formulate goals. Short-term goals, long-term goals. What should we achieve in so and so much time and when will the long-term goal be reached.* | X |  | 6 |
|  |  | High acceptance of the program | *When I look at these areas, I also see a general willingness to use digital offerings, which must be there in any case.* | X |  | 3 |
|  |  | Group exercise sessions | *They often say they want to train together in a group and feel more motivated if they have fellow sufferers around them with whom they can train together.* | X |  | 1 |
|  |  | Family support | *How am I supported by my family. X, I have a background that simply supports me. Where I also have a bit of a safety net at home. Who support me on my way* | X |  | 1 |
|  | Personnel’s motivation | Trust/Accept the program | *… the exercises are demonstrated in a reasonable way and we know, okay, he can also do this on his own, because in principle no correction is possible in this sense.* | X |  | 2 |
|  |  | Involvement in development process | *Involvement. At the VBG (statutory accident insurance provider), it really is the case that we now have a great involvement in the program design.* | X |  | 2 |
|  |  | Interest in the well-being of the patient | *That's why it's certainly exciting for us to see. And from my point of view, the motivation is definitely there to also supervise such aftercare. Simply out of interest to see if it works or if he can participate in the sport he did before? But more on the level of being interested in how the patient is doing after rehab.* | X |  | 1 |
|  |  | More diversity of tasks | *Now, from the point of view of the practitioner's workplace, it's a much broader spectrum and goes with the zeitgeist of digitalization and digital positioning. Because it will come, and it will become more and more and it's great to be able to offer something as multifaceted as that. So, workplace design, instead of doing conventional measures all the time.* | X |  | 1 |
|  | Availability of necessary hardware to patients |  | *What the patient needs is a technical device, a laptop or tablet or, yes, a PC. Some kind of mobile device that he can handle well.* | X |  | 4 |
| Technical | High Usability | Easy to use/time saving | *For me, it is very important, and this is actually one of the most important things, that an app like this is, let's say, very easy to use, both for the user, the patient, and the therapist. Because the therapists, if they have to be instructed in a very complex way, that is very time-consuming, which is now quite difficult.* | X | X | 14 |
|  |  | Clear layout | *I don't know what exactly is meant by user-friendliness. I think it has to be clear, there must not be too much information, the patient has to know in principle beforehand, okay, these are now my three, four areas that I have to deal with.* | X |  | 4 |
|  |  | Easy access | *I think that access to it should be as free of complications as possible.* | X |  | 2 |
|  | Data security | The personnel feel that their data is secure | *That is data protection conform, so where also no doctor, no physiotherapist, so no affected person must be afraid that there is something that does not fit under data protection law.* | X |  | 4 |
|  |  | The patients feel that their data is secure | *Well, I mean, in psychotherapy, very confidential things are discussed, and I think it's simply important that the patients know, for example, that this is protected by data protection.* | X |  | 2 |
|  | High functionality of the program |  | It should be guaranteed, which of course is probably difficult, but that there is simply a stable technical connection, so that there are no server problems. | X |  | 5 |
|  | Anonymity of online psychotherapy | No fear of stigmatization | *But if you now imagine that the patients have to go to a clinic and then someone sees them going to the psychotherapy office. That's the advantage of Clickdoc, of course, if they're so afraid of stigmatization that it's more anonymous to come to the session.* | X |  | 1 |
|  |  | For patients with preconceptions about psychotherapy | *Or even people who have reservations about psychotherapy, I think it's a good way to make the first contact.* | X |  | 1 |
| Environmental and Organizational | Having regular in-person therapy meetings during telerehabilitation aftercare |  | I think the combination is quite good, that you don't just do digital. Because that's all well and good, but the trainer doesn't see. There are so many things where you do not stand correctly or where you still have to pay attention because you cannot see yourself and you cannot correct yourself. And that's why it would always be good to have analog in a mix with digital, because the correction is then also given. | X | X | 11 |
|  | Same therapist in in-person rehabilitation and telerehabilitation aftercare |  | *First, it is important for me in such a setting that the therapist has cared for the patient himself beforehand. This means that the patient must have been my patient during the phase in which he or she was here, so that I can also care for him or her in the further course of aftercare. If this is not the case, I do the same as a substitute because the actual therapist is on vacation. In that case, it's very difficult for me to assess the situation, because I don't know exactly what kind of person he is.* | X |  | 5 |
|  | The therapists’ work schedule has fixed time periods for telerehabilitation aftercare |  | *That there are really fixed times, that I don't have to somehow think about it myself: "Oh yes, I have to take a look in there again. Instead, it's a little bit predefined.”* | X |  | 4 |
|  | Therapy rooms in clinics with hardware, exercise equipment and Wi-Fi |  | *Where a touchscreen is attached to it, which can be used to control the program. In addition, each workstation has a desktop, which can be lowered, is equipped with small materials such as small dumbbells, Airex cushions, a mat, a stool, a ball. Exercise balls are available and so on.* | X |  | 2 |
|  | Financial support of the patient to buy hardware |  | *Yes, so the question is, do you then assume that the patient has the technology available or is that supported? Then something is also financed for him, so to speak.* | X |  | 2 |
|  | Saving costs |  | In addition, you also save as a company, of course, you have to invest once, as a company, no question, but you also save ultimately costs that still arise here in the house, because in principle you only have one therapist afterwards, maybe two, who then ultimately go into the exchange with the patient. | X | X | 2 |

^a^ Number of stakeholders that discussed the subtheme
