# Supplementary material for: Barriers, Facilitators, and Requirements for a Telerehabilitation Aftercare Program for Patients After Occupational Injuries: Semistructured Interviews With Key Stakeholders
Source: JMIR Form Res. 2024 Nov 8;8:e51865. doi: 10.2196/51865 (PMC11584548; doi:10.2196/51865)
Supplement: Multimedia Appendix 4 [file formative_v8i1e51865_app4.docx]

**Multimedia Appendix 4.** Expected barriers of the telerehabilitation aftercare program for patients after occupational injuries.

| Type of barrier | Theme | Subtheme | Example quotes | Stated by | | Number of Stakeholders^a^ |
| --- | --- | --- | --- | --- | --- | --- |
|  |  |  |  | Personnel | Patients |  |
| Individual | Low digital affinity and skills | Low digital affinity of patients | *If this is someone who is not so digital affine, then the motivation to carry out such a digital follow-up is certainly not so high, I can imagine.* | X | X | 8 |
|  |  | Low digital skills of older patients | *Of course, it also depends on the patient clientele, the younger ones are sometimes a bit more open to it and the older generation less so, of course they already have these problems with the technology.* | X | X | 8 |
|  |  | Low digital affinity of healthcare professionals | *Because there are also practitioners who are over 60, and they don't have such good PC skills. And it is also the case that you have to have stamina and keep doing internal exercise.* | X | X | 5 |
|  | Missing language skills of the patients |  | *We already have a larger proportion of insured persons who have difficulties with the German language.* | X |  | 6 |
|  | Low Cognitive Skills of the patients |  | *It is certainly excluded for people with cognitive impairments. So, when I think of patients after brain trauma, where the short-term memory is affected or the ability to act, and sequence planning or interaction ability.* | X |  | 5 |
|  | Patients are not yet able to perform exercises unassisted |  | *That's why we are mostly on therapists side. Yes not most of the time but often not quite in line with the opinions of doctors. How quickly they should return to work. So that is partly horrendous in my eyes. And I think it's questionable who can judge whether they're ready to train themselves. Or whether they still need closer supervision, personal support* | X |  | 3 |
|  | Patients home not suitable | Missing Hardware | *How far you have been involved with the digital services, how you stand financially, and perhaps how you are able to purchase the items.* | X |  | 2 |
|  |  | Missing exercise equipment | *So, the exercise equipment that is given in an institution, if I were on site, that is definitely not given here at home* |  | X | 2 |
|  |  | No personal space | *That is also-, our patients are special now, we have a large part of Bau-BG, I already said, they live in accommodations of ten, twenty, and there is of course with the care is not quite so ideal. They don't all have the best home conditions. And so are a large proportion of the patients.* | X |  | 3 |
|  | Lack of trust and acceptance | Low acceptance/trust by patients | *Yes, of course the acceptance was relatively low. So it was maybe, yes, two, three percent of our patients who wanted to take advantage of this offer.* | X | X | 7 |
|  |  | Low acceptance/trust by personnel | *It was important to us that we train the therapists comprehensively so that they don't have the feeling that we are taking work away from them that they just like to do. Exactly. So that's where the greatest skepticism came from, along the lines of: "That can't work at all, and how is that supposed to work well with videos?* | X |  | 2 |
|  |  | Telerehabilitation and face-to-face meetings are not considered equal by patients and personnel | *Despite possible digital video or photo option that can be done there to monitor what a patient is doing, it is not as close as the in-person exercise.* | X | X | 4 |
|  | Less social interaction | Missing face-to-face interaction | *I believe that many simply lack this direct exchange with the therapist, i.e. that therapeutic relationships somehow take place, which is very, very important for many.* | X | X | 5 |
|  |  | Lower adherence | *I would also say to myself that perhaps compliance is also lower, so somehow more impersonal, although with a picture, but it is more impersonal.* | X |  | 1 |
|  |  | Therapeutic relationship cannot be established | *Is it of course easier in a personal conversation to find a good relationship, working level to discuss the sensitive issues or to discuss constructively.* | X |  | 4 |
|  | Motivation | Low self-motivation/motivation | *Of course, you often hear from patients that they don't have the motivation for digital aftercare. I think that also plays a big role.* | X |  | 5 |
|  | Fear of being replaced | Therapists fear they will be replaced by telerehabilitation | *Of course, we as therapists always have a bit of-, there are still the people who are also my age who then think, oh God, our work is being taken away.* | X |  | 2 |
|  | Exercises are not work-related |  | *So in itself, the job-related content is missing. They have a lot of great exercises for extremely fit patients, including a lot of plank variations and squat variations, some of which I think really healthy people can hardly do. However, the reference to the profession, to everyday life, is very lacking.* | X |  | 4 |
|  | Exercises are too difficult |  | *So, they have a lot of great exercises for extremely fit patients, so also a lot of plank variations and squat variations that I think really healthy people can hardly do to some extent.* | X |  | 1 |
| Technical | Internet connectivity problems | Slow internet speed in rehabilitation clinics | *Then, as we can see right now, the Internet connection is also critical for us. The wi-fi connection can be very poor or very, yes, irregular.* | X |  | 6 |
|  |  | Slow internet speed in patients’ home | *It really depends on what kind of connection they have close to home. I have a patient where we can't do that at the moment because they simply have such a poor Internet connection.* | X | X | 6 |
|  | Privacy concerns or issues | Data protection regulations hinder the use of programs | *We as VBG (insurance provider) do not offer a program like Zoom, so we are not allowed to take Zoom, which is just so secure in terms of data protection law that we can talk about medical or personal data. Unfortunately, this is still not possible.* | X |  | 2 |
|  |  | Patients’ concerns about data privacy | *Because it's also simply about the so-called confidentiality obligation, yes, about data protection. That would of course be very important to me in this respect. If I close the door today during my analog visit, this morning in the clinic, then I assume that I can tell this room what my injury concerns. And that stays in that room. It is not brought to the outside. So nowadays, as a 60-year-old, I have a bit of a hard time with that every day. And I think that everything is taken very lightly these days* |  | X | 1 |
|  |  | Personnel concerns about data privacy | *Especially because, of course, with the video, that could also somehow, yes, you can somehow save it at home, you can copy it, or at least the fear is there that that happens. Or you could be nailed to it. So you have to be very sure of what you say, of course. Or what you pretend to say. And accordingly there is also a hurdle with the therapist, I think.* | X |  | 1 |
|  | Functionality problems of telerehabilitation program |  | The linchpin is, of course, the high functionality of any technology. | X | X | 9 |
|  | Elements of therapy cannot be performed online | Monitoring how patients perform the exercises | *Both in terms of, let's say, the execution criteria of the individual exercises, where the therapist then always has to check again: Is everything correct? Are there any mistakes creeping in? As well as the motivation* | X | X | 5 |
|  |  | Monitoring if patients perform the exercises | *And if nothing comes up, then we assume that everything is fine. Of course, there is always the danger that people will simply skip the exercise. But then we don't really notice that either.* | X | X | 3 |
|  |  | Screen is distracting | *So I have to somehow get to each other to lie on my fitness mat and at the same time still look at the screen and yet listen to what I should actually do now.* |  | X | 2 |
|  |  | Exposure therapy | *Example trauma confrontation. Or in general, that it's just more difficult to really do exercises. I can instruct them via video, digitally, but-.* | X |  | 4 |
|  |  | Physical examination | *And, a physical examination is pointless over internet anyway.* | X | X | 2 |
|  |  | No haptic | *Yes, on the one hand, the therapy content, i.e. in physiotherapy it is still very hands-on, i.e. the patients' expectations are still such that haptics are required, that we touch, feel, sense* | X | X | 8 |
|  |  | Exercises cannot be tailored to patients needs | *Therefore, there is still a lack of content or optimal content for patients with occupational injuries. This differs to some extent from patients with pension insurance. And there is just the statement of both the senior physician of the BG department and the senior therapist of the BG department, that they say, there is simply still some content missing in the area* |  | X | 1 |
|  | Different digital programs for different health care providers |  | *Health insurance has its own program, BG (**statutory accident insurance) has its own program. And that's what I think sucks.* |  | X | 2 |
| Environmental and Organizational | High Costs | Telerehabilitation provider goes bankrupt | *There are many, many providers on the market who all offer similar apps, aftercare apps. And if I install one in the clinic today, it costs a six-figure sum with setup and everything. And if the company goes bankrupt next year, we're back to square one. And everyone has a hard time deciding which provider to choose, because they are all relatively new and no one knows who will stay with them longer.* | X |  | 2 |
|  |  | Invest in Wi-Fi expansion | *We have had to invest an incredible amount here. The WLAN alone was expanded. And if you do it professionally, you quickly run into a five-digit euro range that you have to spend on it.* | X |  | 1 |
|  |  | High costs for telerehabilitation provider | *It also has to be said that equipping this entire room with these touchscreens is of course not at all inexpensive. This small material is the least of the problems. Also the costs that telerehabilitation providers demand for the app and for the provision and the like are also not insignificant.* | X |  | 1 |
|  | Implementation problems | Not fulfilling the time schedule | *If we start a project like this now, or if we say here in the administration: "We want to tackle this now," and if it then takes another year and a half until we actually implement it, the willingness to do it or participate in it simply decreases, or also the interest in it.* | X |  | 2 |
|  |  | Decision-makers change their minds during the implementation process | *Of course, there is often a problem with implementation, because you would probably have to spend a lot of money on it, and you find that sometimes the cost-benefit factor is not so high and you can't really convince the people who are going to make the decisions.* | X |  | 1 |

^a^ Number of stakeholders that discussed the subtheme
