# Supplementary material for: Barriers, Facilitators, and Requirements for a Telerehabilitation Aftercare Program for Patients After Occupational Injuries: Semistructured Interviews With Key Stakeholders
Source: JMIR Form Res. 2024 Nov 8;8:e51865. doi: 10.2196/51865 (PMC11584548; doi:10.2196/51865)
Supplement: Multimedia Appendix 5 [file formative_v8i1e51865_app5.docx]

**Multimedia Appendix 5.** Requirements of the telerehabilitation aftercare program for patients after occupational accident.

| Theme/Value | Attribute/Persuasive Element | Requirement | Requirement type^a^ | Example quotes | Stated by | | Number of Stakeholders^b^ |
| --- | --- | --- | --- | --- | --- | --- | --- |
|  |  |  |  |  | Personnel | Patients |  |
| Tailored program | Tailored physical exercises | **Tailored exercise plans via videos**: Patients receive audiovisual instructions on how to perform the exercises tailored to them, frequent difficulties in performing the exercises, and common evasive movements. Exercise plans are tailored to the type of occupational injury, functional status, patients’ goals and job requirements of the patients. | Contents | *In any case, that you are shown the exercise once. What difficulties there may be or what to look out for. Which evasive movements are common. How to counteract them.*  *So even there, a roofer needs a different rehab program than someone who sits at a desk. And also, a different one than a crane operator, because he has to climb ladders and then sit all day.* | X | X | 15 |
|  |  | **Film tailored exercise videos:**  Therapists can film and share their own exercise videos tailored to the needs of an individual patient. These videos can be important if the video platform does not contain videos that relate to a specific job requirement of the patient. | Functional | *I think it helps a lot more if you shoot a short video of yourself. Or of the therapist. And do it that way…We just do that because we see that it's practicable. It helps patients much, much more.* | X |  | 1 |
|  | Tailored information on injury | **Information on how to cope with injuries and nutrition information:** Patients get access to tailored information on how to cope with their injury in everyday life and nutrition information. | Content | *How can the patient change his behavior so that he can cope better with the injury in everyday life? Or how can he adapt his relationship, i.e., his home, his job, so that he can cope better with the injury?*  *This continues with the diet. If you move or can move in a restricted way, you have to pay a little more attention to your diet.* | X | X | 6 |
|  | Personalized relation exercise/ be in charge | **Selection of relaxation exercises:** Patients can choose between different relation exercises | Content | *Okay* *these are the relaxation exercises that we offer and just choose them yourself. To make it a little easier with this personalization.* | X |  | 3 |
|  |  | **Patient adjustment of exercise intensity:** Patients can adjust exercise intensity within a range predetermined by the therapist. | Functional | That they can perhaps say a little bit themselves, ok, ... I set the degree of difficulty maybe just a little bit higher and do now instead of somehow five repetitions per side, I now do ten repetitions per side or something. | X |  | 1 |
| Social interaction | Social interaction/ therapeutic relationship | **Psychotherapy in real-time:** Patients get psychotherapy via real-time video call. | Content | *I think I (psychotherapist) can really treat him via video conference.* | X | X | 7 |
|  | Social interaction/ exercise monitoring | **Digital and in-person rehabilitation aftercare in a blended care approach:** Patients receive a combination of telerehabilitation with regular in-person therapy sessions. The regular face-to-face therapy session, would, for example, allow monitoring of exercise execution. | Content | *In other words, I can well imagine that we could say something like what we are now planning with the pension insurance companies, a kind of hybrid treatment "two days on site, two days digitally at home", which I think would be a good mix.* | X | X | 6 |
|  | Social interaction/exercise monitoring | **Real-time video call**: Patients, health care providers and rehabilitation mangers can communicate via real-time video call. The real-time video calls enable patients to participate in individual and groups exercise therapy, psychotherapy, and medical consultation (with their physician). Physical therapists can guide, observe, and correct patients' exercise execution. Additionally, psychotherapists can share his screen during the video call to share his notes with the patient. | Functional | *I can also imagine that in the context of a video conference, because I think that, because you always already have a certain image and a certain facial expression and body language, different than on the phone. I can imagine this already. Until now, we have been prevented from doing so because of data protection laws. But why not? I think that if psychotherapy can take place digitally, then counseling can also take place digitally.* | X | X | 15 |
|  | Praise/Reward/ Social Role | **Praise for completing exercises**: Patients receive written praise from the program for completing exercises. | User Experience | You receive a massage. That is said: Here, you've done great, you've now done your exercises for seven days or achieved something special. So now not somehow-, no material value, but simply such an incentive. | X |  | 1 |
|  | Establishing a therapeutic relationship / Social interaction | **Same therapist in** **in-person rehabilitation and telerehabilitation aftercare:**  The same therapist guides patients through the in-person rehabilitation, trains them to use the telerehabilitation program toward the end of in-person rehabilitation and provides support to patients in telerehabilitation. | Work context | *First of all, it is important for me in such a setting that the therapist has cared for the patient himself beforehand. This means that the patient must have been my patient during the phase in which he or she was here, so that I can also care for him or her in the further course of aftercare. If this is not the case, I do the same as a substitute because the actual therapist is on vacation. In that case, it's very difficult for me to assess the situation, because I don't know exactly what kind of person he is.* | X | X | 5 |
| Integration in therapists work context | Ease to use program/time saving | **Exercise plans are easy to create and adjust:** Therapists can choose from pre-designed exercise plans. The pre-designed exercise plans depend on the type of occupational injury, functional status, goals and job requirements of the patient. Therapists can change the frequency and intensity of exercises and make exercise plans repeat themselves with a single adjustment. | Functional | *You would have to have a pool probably of exercises and then someone would have to be able, like a physiotherapist, to choose: These exercises are interesting and relevant for this patient. And then you could do them in some kind of app, individually tailored: Kathrin now has such and such injuries. She can do these and these exercises.*  *That you can also adjust that in any case. It shouldn't be a blanket rule, always only five times a week. But that we or the therapists who give it to the patient can then also adapt it accordingly to the frequency.* | X | X | 13 |
|  | Concern about overtime/ fixed time | **Fixed time for telerehabilitation work:**  Health care professionals get fixed time periods where they can do telerehabilitation work. For example, to read patients comments on their exercise execution, give feedback and create exercise plans. | Work context | *And those therapists, well, regular therapists, that's what we get a certain time of the week to devote to it.*  *Of course, this is also a time issue, because the therapist is in treatment and doesn't have that much time. Time must be given for this.* | X |  | 7 |
|  | Telerehabilitation work is valued by clinics/ work satisfaction | **Digital Workstation**: Therapists get offices with modern computers or mobile devices and a fast internet connection. Physical therapists need high-definition cameras, enough space and exercise equipment to demonstrate exercises in real-time video calls. | Work context | *Providing human resource and structural resource would then mean setting up workstation design and email address and setting up CURALIE (telerehabilitation program) on every possible therapeutic computer workstation and equipping existing laptops with the system as well, which has already been done.*  *The premises, of course, that you have space, not only a PC, but perhaps also a mat where you can actively show and demonstrate certain exercises. That means, of course, that the room needs a certain size.* | X |  | 13 |
| User friendliness/ Usability | Easy access | **Easy Access of the program:** The program can be easily accessed from the computer or mobile devices. If the program will be available as a mobile application, then it should be available for IOS and Android and should work on older hardware. | Functional | *Yes, so it would be important that it would run on all devices, right? So, it would work on mobile devices like tablets or cell phones or smartphones or even on computers? Especially for older people or if the phone is too old or something.* | X |  | 6 |
|  | Multilingual program | **Program is available in different languages:** The rehabilitation aftercare program is available in different languages. | Functional | *“Berufsgenossenschaft der Bauwirtschaft” mainly, so a lot of people from the construction industry. We have problems with a large proportion of our patients not speaking German, so an app can be very helpful if it has several languages. So that would also be a very important requirement for me.* | X |  | 2 |
|  | Reminders | **Daily reminders:** Patients are reminded daily to perform their exercises. The reminders can be sent via e-mail or directly via the program. | Functional | *And they have to be contacted on a daily basis, either via e-mail or directly via the program, whether they have already done their exercises today, what have you done?* | X |  | 1 |
|  | Easy to use program/ tunnelling | **High Usability:** The telerehabilitation aftercare program should be clear and self-explanatory for patients and personnel. Font sizes and contrasts are set to ensure that they are acceptable to elderly patients. The program should find a balance between giving the user functional possibilities and overloading him with choices. A possible solution to this challenge was to have the program guide users step-by-step through a predefined sequence of actions called tunneling. Tunneling removes all unnecessary functions that can possibly distract users’ attention from completing the process. | User Experience | *That it is really clear, descriptive and best of all self-explanatory in terms of usability.*  *Okay, exactly. So that the system guides you step by step through the individual tasks that I have to complete. And be it as a doctor or as a therapist or as a patient. So, each of these roles probably has different tasks.* | X | X | 14 |
|  | Stakeholders’ involvement | **Iterative improvement of the program:** Personnel has a contact person at the telerehabilitation provider to whom they can turn for problems and suggestions for improvement. The concerns are discussed and, if necessary, the program is adjusted. | Service | Yes, so we definitely have a super good contact person who is available for us 24/7 and takes requests. Even if we have wishes, ideas, which should be implemented. | X |  | 1 |
|  | All in one System | **Patients’ medical records can be accessed:** Patients can read his personal medical record. | Content | Basically, the patient image should be stored there so that you can also access it via this app | X | X | 3 |
|  |  | **Submit and track status of applications and resolutions:** Patients can submit and track status of applications and resolutions to the statutory accident insurance. | Functional | Where they can submit applications, where they can get a response, what the processing status is, where they can track that it's been seen, where they can also communicate. | X | X | 2 |
| Monitoring | Exercise monitoring/Feedback/ Social Support | **Asynchronous communication features:** Patients and health care professionals can communicate with each other via asynchronous communication features. Patients can give comments on their exercise session via a chat function or voice massage, share photos, for example, of swollen limbs with their physician or contact their rehabilitation manager about problems at work or in the social context. Health professionals receive an overview of all their received messages when he logs into the program and can then reply. | Functional | *Is it visible externally in any way? Somehow a photo documentation, if it would include that, that would also be good. That a patient can write, "Look, this is what my knee currently looks like, it's a little bit thicker, it's reddened-." And such information can also get to the therapist.*  *If the patient then does an exercise at home, that you somehow have a tool with which you can regularly follow up if he has questions, for example, about certain exercises that you have instructed him to do, in the form of a chat or whatever, that you can work on it together.* | X | X | 10 |
|  | Exercise Monitoring/ Self-Monitoring | **Motion tracking and feedback in real-time:**  Patients exercise execution is evaluated via motion tracking. For example, is the range of motion sufficient for flexion and extension of the wrist? Patients receive feedback on their execution in real-time. | Functional | *There is also the possibility to show movements three-dimensionally via camera and electrodes on the body. So, it is with the Nintendo Wii or I don't know which consoles there are, to simply build in another safety feature.* | X |  | 4 |
| Digital skills and affinity | Rehearsal/ improve digital affinity and skills | **Recurrent trainings of** **personnel:** Personnel receives recurrent trainings on how to use the telerehabilitation aftercare program by personnel of the telerehabilitation provider. | Service | *All the PCs, setup, training of staff is important. And recurring training, I notice that.* | X |  | 8 |
|  | Technical Support/ improve digital affinity and skills | **Technical support for personnel and patients:**  Personnel and patients can call a technical support hotline in case of technical problems. | Service | *It has to be easy to use, it has to have a hotline where you can call immediately if something is wrong, which is of course always very important with such things.* | X |  | 7 |
|  |  | **Patients are introduced to the digital program during rehabilitation:**  At the end of the rehabilitation, the patients receive a content and technical introduction to the aftercare program from trained therapists. | Service | *This app must be used right from the start in the clinic with the patient and the practitioner. If he only starts at home, then I don't think it will be accepted.* | X |  | 5 |
| Privacy concerns | Data security | **Secure Data sharing among and within personnel and patients:** Data sharing within the program fulfills the General Data Protection Regulation of the European Union. | Service | *Optimally, also the possibility to pass diagnostic findings back and forth in a data protection-compliant manner.* | X |  | 6 |
| Costs | Financial Support | **Patients receive financial support to buy hardware:** Patients that do not have the necessary hardware to participate in the telerehabilitation aftercare, receive financial support to buy the hardware. | Service | *Yes, so the question is, do you assume that the patient has the technology at his disposal or is that supported? Then something is also financed for him, so to speak.* | X |  | 1 |

^a^ Classification based on van Velsen et al . [1]: Functional: requirements specifying technical features and prescribe the kind of technology; User experience: requirements specifying how the technology should interact with the user; Service: requirements specifying desired services surrounding the technology; Work context: requirements specifying how the technology should be integrated into the existing work context and routines; Content: requirements specifying the content that needs to be communicated via the technology

^b^ Number of stakeholders that discussed the subtheme.

1. Van Velsen L, Wentzel J, Van Gemert-Pijnen JE. Designing eHealth that Matters via a Multidisciplinary Requirements Development Approach. JMIR Res Protoc. 2013 Jun 24;2(1):e21. PMID: 23796508. doi: 10.2196/resprot.2547.
